# Internet Addiction in the Web of Science Database: A Review of the Literature with Scientific Mapping

**DOI:** 10.3390/ijerph17082753

**Published:** 2020-04-16

**Authors:** Antonio-José Moreno-Guerrero, Gerardo Gómez-García, Jesús López-Belmonte, Carmen Rodríguez-Jiménez

**Affiliations:** Department of Didactics and School Organization, University of Granada, 18071 Granada, Spain; ajmoreno@ugr.es (A.-J.M.-G.); gomezgarcia@ugr.es (G.G.-G.); jesuslopez@ugr.es (J.L.-B.)

**Keywords:** scientific production, bibliometric analysis, scientific mapping, addiction, internet, Web of Science

## Abstract

Information and communication technologies (ICT) is a major element of today’s society with great potential that can offer both advantages and disadvantages. Addiction to the Internet and social networks is a growing problem in all age groups. Education is the context in which to work and train in the correct use of these media. The objective of the study focuses on knowing the scientific production and the performance of the concepts “addiction” and “internet” (ADIN). A bibliometric methodology complemented with the scientific mapping technique was followed. Different processes related to the quantification, analysis, evaluation, and estimation of scientific documents were carried out. The literature was analyzed by specific programs such as SciMAT, Analyze Results, and Creation Citation Report. The unit of analysis was specified in 5644 scientific publications extracted from Web of Science (WoS), belonging to the period of years between 1996 and 2019. The results showed that the evolution in the study of the addiction to the Internet is constant and continuous, with articles in English being the most used means to present the information on the part of the investigators. In addition, the subject of study was based on time, given that the coincidence of key words between the periods analyzed was high. In conclusion, the importance of promoting healthy living habits that include responsible use of the Internet are discussed.

## 1. Introduction

Today’s society is increasingly focused on technology and therefore people are progressively more dependent on it, particularly with regards to the Internet [1,2]. Mobile phones and different technological devices have made the world of video games and online shopping more accessible to everyone, making markets even more desirable and broad than before [3]. 

The Internet and the different social networks have become popular instruments that are currently used to communicate and exchange information, among the most outstanding actions [4]. The way in which people interact with each other, entertain themselves, and consume through the media has been modified through the Internet [5]. However, as more people connect to multimedia devices to share information and make use of the possibilities they are offered, cases of media addiction are growing at a dangerous pace [6,7]. These addictions have grown significantly over the last decade [8], surpassing other types of addictions. 

There is a great deal of scientific evidence showing how there is a serious risk associated with excessive use of the Internet and everything that derives from it [9], where, in addition, personal characteristics, gender, socio-cultural differences [10], and biological factors could play an important role in the appearance of an addiction to these media. The literature reveals an association between the Internet and social networks and an increase in so-called techno-stress [11,12,13].

The constant use of the Internet and social networks leads to an addiction and obsession, which results in negative behavior [14]. The consequences of these habits regarding the excessive use of different smart devices and the Internet can affect both the physical and mental health of users [15]. Issues such as anxiety, depression, impulsiveness, and aggressive behavior can also result or be increased from uncontrolled use [16]. Those with internet addiction have higher levels of depression, anxiety, and impulsivity than those without such an addiction [9].

Numerous studies address internet and smartphone addiction, regardless of the age of those suffering from it [17] or even the field in which they are trained or work [18]. In addition, the literature contains relevant research that addresses this type of addiction and its relationship to the educational setting [8,19]. Technology, the Internet, and the impact of the digital world have a great influence on the student body and the development of their personality [20] and health [21]. 

At present, students, regardless of their educational stage, are one of the groups most closely related to everything related to ICT and its use for different purposes, such as socialization or proper academic development [22]. Experts on the field state that those students who use the Internet for purposes that go beyond simple socialization through the different social networks and video games have a better level with regard to problems related to the use of this technology [23]. The exclusive use of these media for playful or socializing purposes without an educational perspective or previous training in the correct use and handling can lead to problems such as sexting [24], cyberbullying [25], or video game addiction [26]. The consequences derived from these phenomena can affect the academic, social, and emotional spheres. 

In this respect, from the educational field, mechanisms of prevention and action should be established in the face of these new existing trends. Therefore, it is necessary to educate in terms of the correct handling of the devices at a technical level, but also at a regulatory level, where the self-regulation of certain behaviors towards these devices is promoted, always extracting the maximum potential for all the areas of the life of the students, including their academic life [27]. The objective from the educational point of view should be to form digitally competent citizens who have positively developed their ethical, literacy, participatory, and critical aspects towards technology and the use of the Internet [28,29]. 

In recent times, there have been constant publications that analyze ICT, as well as the Internet and its relationship with education [30,31], as the tendency of these topics is to increase and acquire more relevance in terms of research. On the other hand, studies approached from bibliometrics, such as the one presented in this paper, are in recent times a constant that addresses a multitude of topics of incidence, such as medical research [32], virtual reality [33,34], or the area of sports [35].

## 2. Justification of Study

In the research presented, the concepts “addiction” and “internet” (ADIN) are analyzed in the impact scientific literature collected from the Web of Science (WoS) database. Web of Science is an online platform containing databases of bibliographic information and information analysis resources that allow the evaluation and analysis of research performance, especially in the field of social sciences. In addition, it presents a series of analysis tools that allow specific and concrete searches to be carried out [36]. This study acquires a bibliometric nature with the purpose of analyzing the publications by means of a scientific mapping developed as a result of various bibliometric indicators and a dynamic and structural evolution of the aforementioned terms.

To carry out and follow a study model validated by experts in this field of research, other impact investigations taken from the Journal Citation Reports were taken as a reference (JCR) [36,37].

This study focused on the evaluation of the trajectory and evolution of the mentioned concepts of the main WoS collection. Initially, a search was carried out in such a database to know the status of the terms issue and, in addition, to check if there were any studies of similar characteristics to the one presented in this work. After the search phase, no publication was found that analyzed both terms with a scientific mapping, and thus this study acquired an exploratory nuance about the state of the art in impact literature. Therefore, this study helps to increase the findings and reduce the gap found in the literature concerning the relationship between “addiction” and “internet”. Therefore, the results obtained will acquire a relevant value as they will contribute to the advancement of science, in addition to serving as a basis for successive research.

## 3. Materials and Methods

### 3.1. Research Objectives

The general objective of this study was to analyze the scientific literature on ADIN indexed in the WoS database. This general objective is articulated in the following specific objectives:

- To determine the performance of publications on ADIN in WoS.
- To decree the scientific evolution of ADIN in WoS.
- To find out the most incident issues about ADIN in WoS.
- To identify the most influential authors in ADIN in WoS.

The research questions posed are

- RQ1: What is the performance of ADIN publications in WoS?
- RQ2: What is the scientific evolution of ADIN in WoS?
- RQ3: What are the most frequent problems of ADIN in WoS?
- Q4: Who are the most influential authors of ADIN in WoS?

### 3.2. Research Design

The research methodology adopted in this study to achieve the proposed objectives was bibliometry. This choice is justified in the scientometric potentialities linked to the actions of searching, recording, analyzing, and predicting scientific literature [38]. Similarly, the guidance and considerations of experts in this type of study were followed [39].

Specifically, this work was based on the analysis of co-words [40] and various bibliometric indicators such as the h-index, taken as a reference, in addition to other indices (g, hg, q2) [41]. This gave rise to the creation of node maps whose interpretation determined the performance and position of conceptual subdomains alluding to ADIN. In addition, the analytical treatment delimited the thematic development of these concepts in WoS [42]. From this database, we used the complete record in order to obtain as much information as possible, in order to have the most relevant data to perform the co-word analysis [43].

### 3.3. Procedure and Data Analysis

This investigation was carried out following various actions that specified the following procedures: (a) selection of the database (WoS), (b) determination of the keywords (“addiction” and “internet”) after consultation in specialized thesauri (ERIC and UNESCO) and in the information provided in the Special Issue of the *International Journal of Environmental Research and Public Health*, and (c) construction of a precise search equation to obtain significant results (“addiction*” [TOPIC] AND “internet” [TOPIC]) and report documents containing both terms in different metadata (title, abstract, and keywords).

The first search action compiled a scientific volume of 5776 publications. In addition, the entire report was analyzed to eliminate repeated documents, those belonging to the year 2020 and the poorly indexed ones. After this, an analysis unit of 5644 publications remained, as a result of the configuration of various production indicators with their respective inclusion criteria (Table 1).

**Figure 1:**
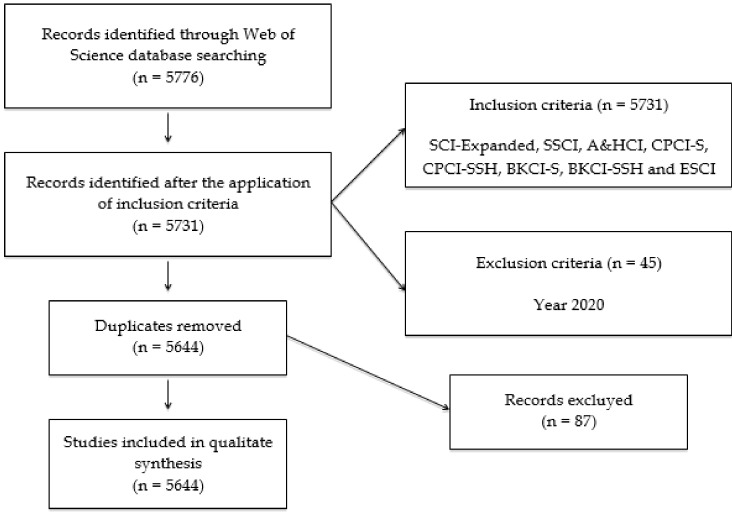
Flowchart according to the PRISMA Declaration.

To improve the understanding and visualization of the different actions carried out, the following flow chart was established following the protocols of the PRISMA-P matrix (Figure 1).

The documents obtained were analyzed with various programs (Analyze Results, Creation Citation Report, and SciMAT). The first two tools are provided by WoS and were used to obtain data concerning the year, authorship, country, type of document, institution, language, means of publication, and most cited documents. For the structural and dynamic development of a longitudinal nature of the scientific volume, SciMAT was used, under the considerations of the experts [44].

With SciMAT, a co-word analysis of the topics was carried out through the following processes:

- Recognition: In this process, various actions were carried out. The keywords of the literature obtained were analyzed (*n* = 10,555). A map of co-occurrence nodes was generated. A standardized network of co-words was developed. The most significant keywords were detected (*n* = 9842), and the themes and concepts with greater predominance were represented with a clustering algorithm.
- Reproduction: In this process, a strategic diagram and a thematic network based on the principles of centrality and density were prepared. In the generated graphs, four regions can be seen: (1) top right (motor themes and highlights), (2) upper left (rooted and isolated themes); (3) lower left (themes to disappear or project), and (4) lower right (underdeveloped and transversal themes).
- Determination: In this process, the evolution of the nodes in certain periods or intervals of time was analyzed. In particular, four periods were defined (P_1_ = 1996-2010; P_2_ = 2011-2014; P_3_ = 2015-2017; P_4_ = 2018-2019), except in the authorship that one that covered the entire documentary volume was established (P_X_ = 1996-2019). The number of matching keywords determined the strength of association between the configured periods.
- Performance: In this process, a set of production indicators associated with inclusion criteria that determine the scientific works that become part of the study were delimited (Table 2).

## 4. Results

### 4.1. Performance and Scientific Production

**Figure 2:**
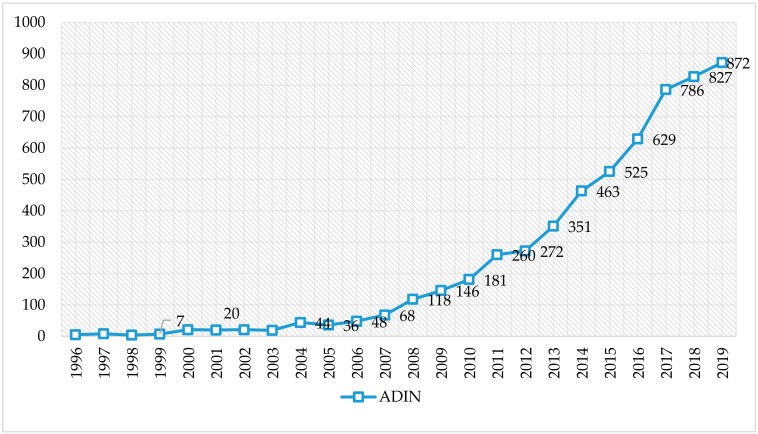
Evolution of scientific production of the keywords “addiction” and “internet” (ADIN) in the Web of Science (WoS).

The evolution of the 5644 documents in the scientific production on ADIN has been constant and continuous over time, showing an exponential growth from its beginnings until the year 2019 (Figure 2). In this sense, and according to the Price law about growth of scientific information, visualization of the distribution of publications on internet addiction doubled in its development 10 years after its first publication. At present, productivity on the subject is in a phase of linear growth.

The language chosen by the various authors for the presentation of the academic results was English (Table 3).

The main area of knowledge in ADIN studies is psychiatry, distanced considerably from other areas of knowledge (Table 4).

The type of document mainly used by the various scientists are articles (Table 5).

Nottingham Trent University is the leading organization in ADIN studies (Table 6).

The most prolific author is Griffinths, M.D., with considerable scientific output on ADIN (Table 7).

The main source of presentation of studies on ADIN are, with similar productions, *Computers in Human Behavior* and the *Journal of Behavioral Addictions* (Table 8).

The country with the greatest interest in production on ADIN is the United States (Table 9).

The reference author for the scientific community in relation to the subject of ADIN, in terms of number of citations, is Davis (2001), followed at a short distance by Morahan-Martin and Schumacher (2000) and Block (2008) (Table 10).

### 4.2. Structural and Thematic Development

**Figure 3:**
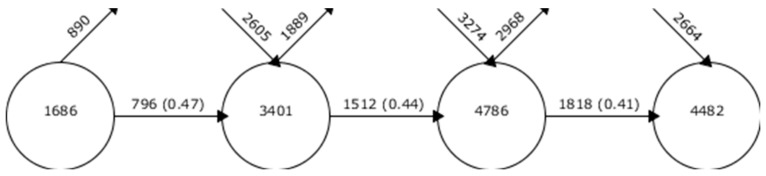
Continuity of keywords between contiguous intervals.

The evolution of keywords shows information about the number of keywords in each of the established time intervals, the number of matching keywords between the periods, and the number of keywords leaving and entering a certain period with respect to another. The studies on ADIN, taking into account the evolution of keywords in scientific production, mark a settled and consolidated line of research, given that the level of coincidence between periods was between 40% and 50% (Figure 3).

The academic performance in the established periods offers the subjects with the greatest bibliometric indicators, using the h-index as the main reference, and completing this information with the g-index, hg-index and q2-index, in addition to the number of citations. On the subject of ADIN, the subjects with the highest bibliometric indicators varied between periods. In the first time period (1996–2010), the topic with the highest bibliometric indicator was “addiction”; in the second time period (2011–2014) it was “prevalence”; in the third time period (2015–2017) it was “depression” and “addiction”; and in the last time period (2018–2019) it was “adolescents”. This shows changes in the interests of researchers in this field of study. We also observed the changes between periods of the topics of greatest interest to researchers, showing the main lines of research in each of the established time intervals (Table 11).

**Figure 4:**
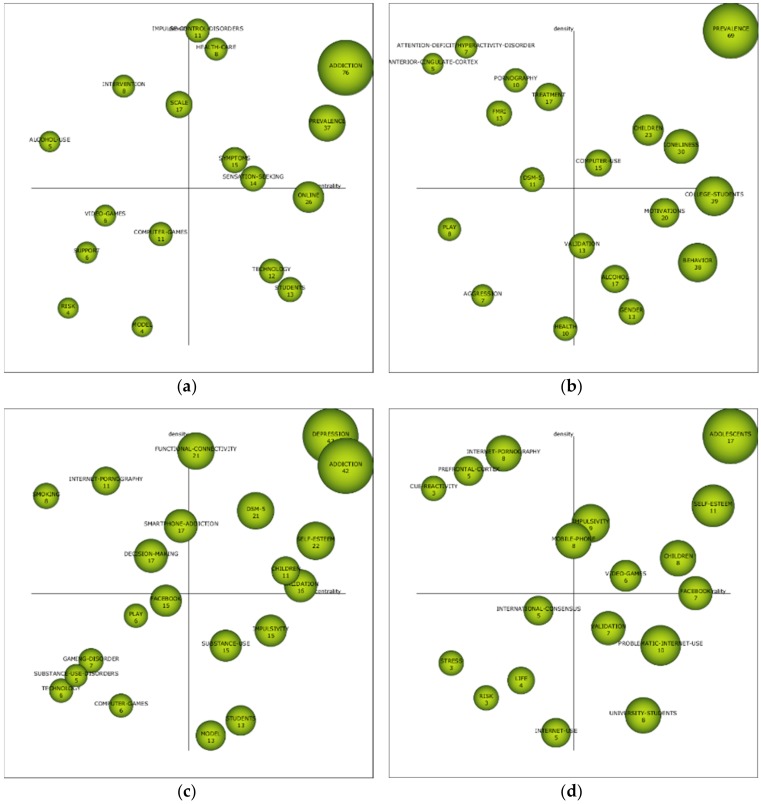
ADIN’s strategic diagram by h-index. Note: (**a**) interval 1996–2010; (**b**) interval 2011–2014; (**c**) interval 2015–2017; (**d**) interval 2018–2019.

The diagrams of the intervals developed show data on the importance of each of the themes in the different periods. For this purpose, a grouping process was developed, according to Callon’s indicators, which assesses the degree of interaction of a network with respect to other networks, from two axes: centrality, which analyses the strength of the relationship of external links with other topics, where it shows the importance of the development of a topic in a field of research; and density, which assesses the internal strength of the network, analyzing the internal links between the key words that are grouped around a specific topic, giving information on the degree of development of a field of study. In the first period (1996–2010), the driving themes were “addiction”, “prevalence”, “symptoms”, “health-care”, and “sensation-seeking”. In the second period (2011–2014), the themes were “prevalence”, “children”, “computer-use”, and “loneliness”. In the third period (2015–2017), themes were “depression”, “addiction”, “DSM-5”, “functional-connectivity”, “self-esteem”, “children”, and “validation”. In the last period (2018–2019), themes were “adolescents”, “self-esteem”, “impulsivity”, “children”, and “video-games”. From this last period, we should also highlight the themes “international-consensus”, “stress”, “risk”, “life”, and “internet-use”, given that their location in the diagram makes them unknown themes, as they may be the next references in the next few years in this field of study, or they may disappear. Generally speaking, the interests of ADIN researchers are focused on aspects related to adolescents, children, frequency, and addiction, as these are the issues that most appear in the motor themes of the diagram (Figure 4).

### 4.3. Thematic Evolution of the Terms

**Figure 5:**
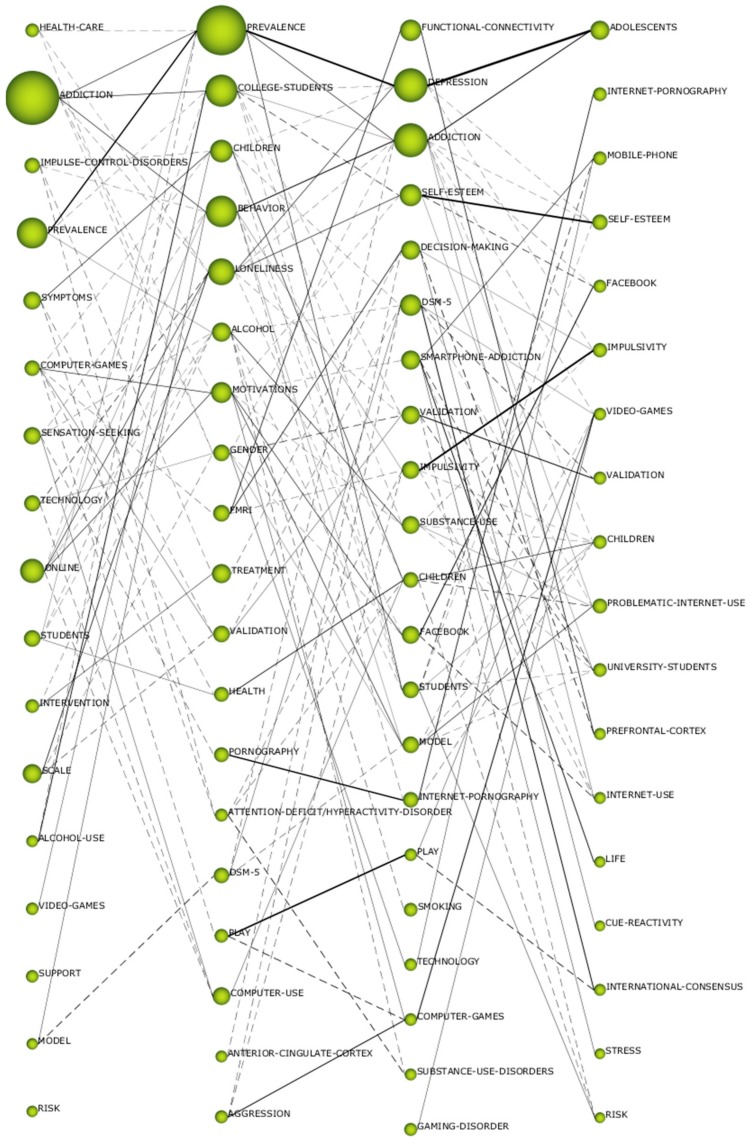
Thematic evolution by h-index.

The thematic evolution offers the strength of the relationship maintained between the themes of the various intervals generated according to the Jaccard index. The development is generated if a theme of a particular interval shares keywords or themes with the subsequent or previous periods. The greater the number of keywords between the two themes of consecutive intervals, the more solid its evolution will be. The possible connections are continuous line, where its connection is thematic, and discontinuous line, whose connection is by keywords. The thickness of the lines shows the strength of the relationship between the themes. The data showed a conceptual gap in the thematic evolution in the ADIN study, as there was no keyword that was repeated in all the established periods. Even so, there were themes that were repeated in at least two or more periods. The timeline with the greatest connecting force was the one established between “prevalence-prevalence-depression-adolescent”, being the basis of the studies on the research topic. There were other strong connections, such as those established between “play-play”, from the second and third period, and “impulsivity-impulsivity” or “self-esteem” from the third and fourth period. In addition, Figure 5 shows that there were an even number of thematic connections and keyword connections. The studies on ADIN had an established basis, focusing mainly on people under 18 years of age and the different pathologies caused by internet use addiction.

### 4.4. Authors with a Higher Relevance Index

**Figure 6:**
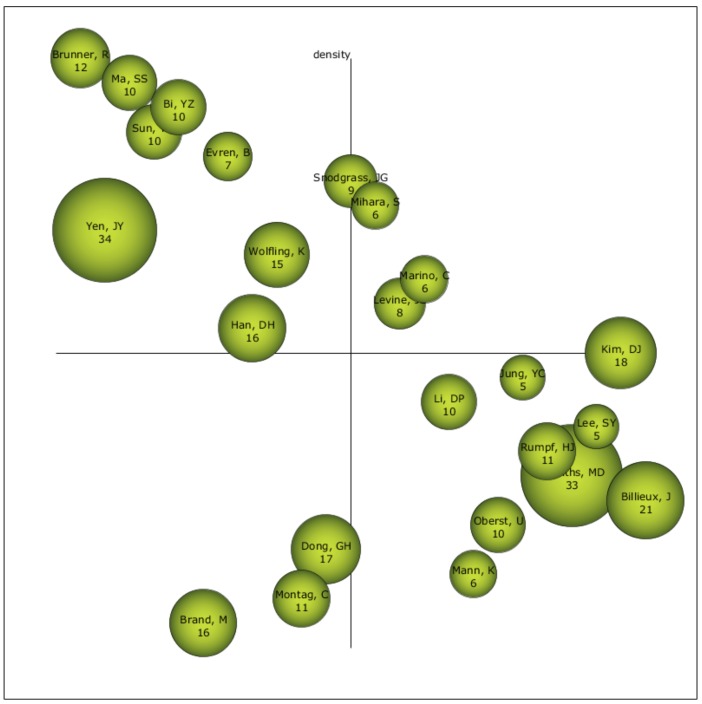
Strategic authoring diagram.

The driving authors on the field of study of ADIN are Mihara, S.; Marino, C.; and Levine, S. In addition, the author that have a great relevance for the scientific community, bearing in mind their h-index, are Yen, J.Y., and Griffiths, M.D., although their location in the diagram placed them as isolated or cross-sectional authors. For the next few years, we should bear in mind the authors Brand, M.; Dong, G.H.; and Montag, C., because their location in the diagram placed them as unknown authors, and they may disappear or be the reference authors in this field of study (Figure 6).

## 5. Discussion

ADIN has become one of the most frequented topics in the scientific panorama over the last years. The arrival of the Internet in people’s lives has brought many benefits, but also risks that citizens must be aware of in order to take preventive measures [9].

The present work aimed to establish a state of the art study on scientific productivity on ADIN in one of the most prestigious databases at present (WoS). The analysis of the data allowed the elucidation of the exponential growth in which the discipline of knowledge finds itself at present, doubling in 10 years from its origin the number of publications and being nowadays in a phase of linear growth [44]. The large percentage of manuscripts found belonged mainly to the psychiatric and psychological area, linked to the treatment of addictions, drugs, and harmful substances, which denotes that this is a global concern that particularly affects the compendium of the health discipline [32]. However, there is also an emerging amount of research concerning the educational branch, which allows us to see that ADIN is beginning to be a concern for experts in the field of education, in order to promote a practice of a prophylactic nature [33].

Likewise, the analysis on the most prolific institutions and authors in the area allowed us to know that the research on ADIN is an object of study by multiple researchers from different parts of the world, with numerous countries being among the biggest producers of scientific works on the subject, and therefore configuring one of the most important risks that reside in the society [1].

In this sense, the co-word analysis provided an understanding of which of the descriptors had the highest academic performance over the years about ADIN, in addition to being able to visualize its evolution over the years. By way of this, it was possible to distinguish how there was an evolution in this issue, from the origin of ADIN to the present, starting with descriptors such as “sensation-seeking“ or “symptoms”, to “loneliness”, “depression”, “self-esteem”, “stress”, “video-games”, or “impulsivity”. Undoubtedly, a negative progression is visualized that began with descriptors that alluded to minor symptoms and disorders, and ended with descriptors referring to risks related to serious disorders or diseases such as depression, social isolation, or serious self-esteem problems. This indicates that there has been an increase in ADIN in society and, therefore, there are publications that refer to this phenomenon associated with this type of disease [9,16].

In sum, the results indicated that, to a greater extent, risks associated with the Internet, such as the consumption of pornography and young people’s addiction to video games and the behavioral disorders associated with them, obtained a considerable density of publication, and therefore these are risks that affect society today [23,24]. In a similar vein, the analysis of the relationship between issues through the Jaccard index allowed us to discern relevant connections between issues, as was the case with the one maintained between “prevalence-prevalence-depression-adolescent”, “addiction-behavior-addiction-adolescent”, or “online-loneliness-self-esteem”. These links allowed us to corroborate the interest of the research community in the risks associated with ADIN over the years, an idea similar to those expressed in previous research [7,17,33,34]. Specifically, the main population under study, according to the works analyzed, are young people, especially adolescents.

Finally, the scientific mapping of authors with a higher index of relevance allowed us to know a considerable compendium of experts in the present on ADIN, which indicates the importance that this topic is gaining within the current scientific panorama, as well as the need for its study and deepening by the research community.

## 6. Conclusions

ADIN is among the most threatening risks to society today and in the future, especially among the younger population, who live more with technological devices continuously, which can lead to severe consequences associated with serious behavioral disorders and psychological diseases.

Through this work, we intended to establish a general state on the productivity and scientific performance of the subject, trying to contribute interesting ideas about the evolution of the subject and about which are the most frequent aspects to be developed by the researchers, who focus their attention on the study of ADIN. All of this information can be used in order to be able to elucidate the working path in the near future.

Among the limitations that comprise this work is the debugging of the data presented in WoS, where in some cases some repeated or unrelated documents are presented. This was also the case in the establishment of the intervals, this being a question of equity, given that the researchers tried to maintain a similar density of manuscripts in each of them. This study focused solely on the WoS database to show the scientific community the state of the art research on the existing literature on the subject. This may also be a limitation of the study. Therefore, as a future line of research, it is proposed that the analysis be replicated in other impact databases such as Scopus. On the other hand, as future lines of research, it is recommended that future authors investigate the search for new risks associated with the excessive use of the Internet, as well as the establishment of effective educational research to help combat and prevent this dangerous phenomenon for present and future generations.

In conclusion, the idea of continuing on the path of promoting healthy living habits with respect to digital health, especially among the youngest people, through a responsible practice to promote a healthy society that avoids coming into contact with the risks associated with the digital world, is stressed.

## Figures and Tables

**Table 1:** Production indicators and inclusion criteria.

Indicators	Criteria
Year of publication	All production except 2020
Language	x ≥ 50
Publication area	x ≥ 300
Type of documents	x ≥ 300
Organizations	x ≥ 70
Authors	x ≥ 60
Sources of origin	x ≥ 100
Countries	x ≥ 400
Citation	The four most cited documents

**Table 2:** Production indicators and inclusion criteria.

Configuration	Values
Analysis unit	Keywords authors, keywords WoS
Frequency threshold	Keywords: P_1_ = (5), P_2_ = (7), P_3_ = (7), P_4_ = (7)
	Authors: P_X_ = (10)
Network type	Co-occurrence
Co-occurrence union value threshold	Keywords: P_1_ = (2), P_2_ = (4), P_3_ = (5), P_4_ = (5)
	Authors: P_X_ = (6)
Normalization measure	Equivalence index
Clustering algorithm	Maximum size: 9; minimum size: 3
Evolutionary measure	Jaccard index
Overlapping measure	Inclusion rate

**Table 3:** Scientific language used in ADIN.

Language	*n*
English	5265
Spanish	119
German	95
French	56
Russian	53

**Table 4:** Research areas.

Research Area	*n*
Psychiatry	1649
Psychology (multidisciplinary)	801
Substance abuse	678
Psychology (clinical)	522
Psychology (experimental)	374
Education educational research	313

**Table 5:** Document types.

Document Types	*n*
Article	4467
Meeting abstract	383
Proceedings paper	347
Review	320

**Table 6:** Institutions.

Institution	*n*
Nottingham Trent University	242
Seoul National University (SNU)	107
Catholic University of Korea	105
Yale University	98
Kaohsiung Medical University	87
Chung Ho Memorial Hospital	76
Johannes Gutenberg Univesrity of Mainz	72

**Table 7:** Authors.

Authors	*n*
Griffinths, M.D.	183
Kim, D.J.	72
Ko, C.H.	67
Yen, C.F.	66
Potenza, M.N.	65
Billieux, J.	63
Brand, M.	60
Wolfling, K.	60

**Table 8:** Source titles.

Source Titles	*n*
*Computers in Human Behavior*	356
*Journal of Behavioral Addictions*	331
*Cyberpsychology Behavior and Social Networking*	168
*Addictive Behaviors*	105
*International Journal of Mental Health Addiction*	105

**Table 9:** Countries.

Country	*n*
United States	1263
China	773
England	511
South Korea	469
Turkey	406
Germany	405

**Table 10:** Most cited articles and main conclusions.

Reference	Citations	Main Conclusion
[45]	881	The model implies a more important role of cognitions in pathological internet use (PIU), and describes the means by which PIU is both developed and maintained. Furthermore, it provides a framework for the development of cognitive-behavioral interventions for PIU.
[46]	593	Pathological users, for pathological internet use (PIU), scored significantly higher on the UCLA Loneliness Scale, and were socially disinhibited online.
[47]	579	This document refers to how the DSM-IV considers internet addiction.
[48]	535	Among the findings are the urgent need to develop research on best practices for treating pain in adolescents, as well as the development of prevention strategies to reduce diversion and abuse.

**Table 11:** Thematic performance.

**Interval 1996–2010**						
**Denomination**	**Works**	**Index h**	**Index g**	**Index hg**	**Index q^2^**	**Citations**
Health-care	12	8	10	8.94	13.86	210
Addiction	238	76	130	99.4	108.19	18,177
Impulse-control-disorder	13	11	12	11.46	25.48	1203
Prevalence	62	37	61	47.51	65.8	5423
Symptoms	20	15	20	17.32	48.68	2216
Computer-games	13	11	11	11	29.1	1328
Sensation-seeking	15	14	14	14	31.3	1547
Technology	22	12	20	15.49	35.16	2060
Online	42	26	38	31.43	43.86	3061
Students	17	13	17	14.87	24.45	848
Intervention	11	8	10	8.94	22.09	523
Scale	21	17	19	17.97	31.4	1488
Alcohol-use	5	5	5	5	10.72	491
Video-games	6	6	6	6	21.77	489
Support	6	6	6	6	21.21	591
Model	5	4	5	4.47	25.14	611
Risk	4	4	4	4	21.82	466
**Interval 2011–2014**						
**Denomination**	**Works**	**Index h**	**Index g**	**Index hg**	**Index q^2^**	**Citations**
Prevalence	519	69	105	85.12	83.89	19,238
College-students	95	39	71	52.62	54.44	5256
Children	44	23	37	29.17	33.57	1384
Behavior	115	38	61	48.15	54.79	4168
Loneliness	81	30	56	40.99	46.8	3339
Alcohol	30	17	30	22.58	28.57	1026
Motivations	28	20	27	23.24	36.06	2048
Gender	27	13	23	17.29	19.42	596
FMRI	15	13	14	13.49	28.62	870
Treatment	26	17	25	20.62	29.15	1038
Validation	16	13	16	14.42	22.8	681
Health	15	10	15	12.25	15.81	228
Pornography	12	10	12	10.95	17.03	406
TDAH	8	7	8	7.48	12.69	481
DSM-5	18	11	17	13.67	26.12	759
Play	10	8	9	8.49	14.7	296
Computer-use	20	15	19	16.88	24.49	724
Anterior-cingulate-cortex	5	5	5	5	16.28	414
Aggression	7	7	7	7	20.32	579
**Interval 2015–2017**						
**Denomination**	**Works**	**Index h**	**Index g**	**Index hg**	**Index q^2^**	**Citations**
Funcional-connectivity	54	21	29	24.68	25.51	998
Depression	571	42	63	51.44	51.44	8642
Addiction	680	42	63	51.44	51.03	9259
Self-esteem	108	22	33	26.94	27.75	1492
Decision-making	35	17	29	22.2	22.58	866
DSM-5	73	21	38	28.25	29.7	1627
Smartphone-addiction	44	17	26	24.74	29.44	1314
Validation	85	16	25	20	21.54	937
Substance-use	52	15	26	19.75	21.21	821
Children	53	15	24	18.97	18.57	763
Facebook	44	11	15	12.85	12.85	331
Students	45	15	22	18.17	18.97	596
Model	47	13	23	17.29	18.73	605
Internet-pornography	43	13	26	18.38	17.66	736
Play	19	11	16	13.27	16.58	361
Smoking	15	6	13	8.83	12.73	179
Technology	10	8	10	8.94	13.56	244
Computer-games	12	6	9	7.35	11.22	126
Substance-use-disorders	8	6	7	6.48	10.39	207
Gaming-disorder	9	5	7	5.92	7.07	68
**Interval 2018–2019**						
**Denomination**	**Works**	**Index h**	**Index g**	**Index hg**	**Index q^2^**	**Citations**
Adolescents	860	17	22	19.34	19.34	2433
Internet-pornography	48	8	13	10.2	11.66	231
Mobile-phone	66	8	14	10.58	12.33	262
Self-esteem	138	11	17	13.97	15.56	544
Facebook	73	7	8	7.48	7.94	200
Impulsivity	68	9	11	9.95	9.95	229
Video-games	82	6	10	7.75	8.49	180
Validation	74	7	9	7.94	7.94	177
Children	112	8	10	8.94	9.8	263
Problematic-internet-use	119	10	13	11.4	11.83	399
University-students	45	8	13	10.2	10.95	222
Prefrontal-cortex	20	5	7	5.92	6.71	60
Internet-use	25	5	7	5.92	7.42	83
Life	16	4	7	5.29	6.93	55
Cue-reactivity	7	3	4	3.46	3.46	20
International-consensus	12	5	7	5.92	6.71	59
Stress	12	3	5	3.87	3.46	33
Risk	16	3	4	3.46	4.58	29
